# SARS-CoV-2 in hospital indoor environments is predominantly non-infectious

**DOI:** 10.1186/s12985-021-01556-6

**Published:** 2021-06-02

**Authors:** Janina Krambrich, Dario Akaberi, Jiaxin Ling, Tove Hoffman, Lennart Svensson, Marie Hagbom, Åke Lundkvist

**Affiliations:** 1 Department of Medical Biochemistry and Microbiology, Zoonosis Science Center, Uppsala University, Uppsala, Sweden; 2 Division of Molecular Medicine and Virology, Department of Biomedical and Clinical Sciences, Medical Faculty, University of Linköping, Linköping, Sweden; 3 Division of Infectious Diseases, Department of Medicine Solna, Karolinska Institute, Stockholm, Sweden

**Keywords:** SARS-CoV-2, COVID-19, RNase A, Environmental sampling, In vitro infection, Viral infectivity

## Abstract

**Background:**

The ongoing SARS-CoV-2 pandemic has spread rapidly worldwide and disease prevention is more important than ever. In the absence of a vaccine, knowledge of the transmission routes and risk areas of infection remain the most important existing tools to prevent further spread.

**Methods:**

Here we investigated the presence of the SARS-CoV-2 virus in the hospital environment at the Uppsala University Hospital Infectious Disease ward by RT-qPCR and determined the infectivity of the detected virus in vitro on Vero E6 cells.

**Results:**

SARS-CoV-2 RNA was detected in several areas, although attempts to infect Vero E6 cells with positive samples were unsuccessful. However, RNase A treatment of positive samples prior to RNA extraction did not degrade viral RNA, indicating the presence of SARS-CoV-2 nucleocapsids or complete virus particles protecting the RNA as opposed to free viral RNA.

**Conclusion:**

Our results show that even in places where a moderate concentration (Ct values between 30 and 38) of SARS-CoV-2 RNA was found; no infectious virus could be detected. This suggests that the SARS-CoV-2 virus in the hospital environment subsides in two states; as infectious and as non-infectious. Future work should investigate the reasons for the non-infectivity of SARS-CoV-2 virions.

**Supplementary Information:**

The online version contains supplementary material available at 10.1186/s12985-021-01556-6.

## Background

At the end of 2019, a novel coronavirus, the severe acute respiratory syndrome coronavirus-2 (SARS-CoV-2) spread around the world, disrupting healthcare systems and affecting the lives of millions of people. On March 11, 2020, the viral pneumonia Coronavirus disease (COVID-19), caused by SARS-CoV-2, got classified by the World Health Organization as the second pandemic of the twenty-first century [1]. As of April 2021, the global count of registered COVID-19 cases has risen to over  146  million [2]. Many reports however indicate that a large number of SARS-CoV-2 infections are asymptomatic and therefore remain undiagnosed [3–8].

Knowledge of transmission pathways is the basis for the prevention of infectious diseases, and fourteen months into the current pandemic, it is still not sufficiently known how each individual infection with SARS-CoV-2 really works. Transmission via inhalation of virus-containing respiratory droplets and via direct contact (contact of mucous membranes with virus-contaminated hands or fluids) has been considered the most significant route of infection, while airborne transmission and persistence of the virus in different environments is still under investigation [9–12]. In regions where the immunization coverage is low, prevention of transmission is the best control strategy to protect against infection. Exposure to SARS-CoV-2 particles in the environment is currently believed to pose a significant risk for infection [13].

In several studies, SARS-CoV-2 RNA has been detected in samples from different surfaces in several hospitals [9–12, 14–16] as well as in hospital ventilation systems [15, 17], indicating that the virus is dispersed through the air. However, attempts to isolate viruses from environmental samples have remained largely unsuccessful or unclear despite the relatively high stability and viability of SARS-CoV-2 on various surfaces [11, 13, 16–18]. In samples from COVID-19 hospitalized patients, SARS-CoV-2 has readily been isolated from nasopharyngeal swabs and occasionally from hospital room air samples collected during the first week after the onset of disease symptoms [19, 20], but a significant decrease in positive results was observed in samples collected in the following days [20]. Despite high RNA loads, no positive isolation from patient samples has been reported after day 10 of onset of symptoms [20–23]. Recovery of infectious virus between 10 and 20 days after the onset of symptoms has only been documented in some individuals with severe COVID-19 symptoms, in some complicated cases by an immunocompromised condition [24].

A recent simulation showed that the relative humidity of indoor air can have a large influence on the potential for viral particle dispersion, indicating an increased risk of SARS-CoV-2 infection under dry conditions [25]. The simulation showed that a relative humidity of less than 30% leads to more than double the amount of aerosolized particles, compared to values of 60% or higher.

Consequently, there is an urgent need to further investigate why SARS-CoV-2 RNA but not infectious virus can be detected in e.g. swabs from hospital environments. This study investigated the presence of the SARS-CoV-2 virus RNA and the viral infectivity of samples collected on various surfaces at different locations in the Uppsala University Hospital Infectious Disease ward in Uppsala, Sweden. The samples were analyzed by quantifying the copy numbers of the SARS-CoV-2 genome by reverse transcription quantitative polymerase chain reaction (RT-qPCR), and a subset of positive samples was subsequently analyzed for the presence of protected SARS-CoV-2 RNA (e.g. undamaged nucleocapsid or whole virions) as opposed to free SARS-CoV-2 RNA fragments using RNase treatment.

## Materials and methods

### Sampling locations and time

**Fig. 1:**
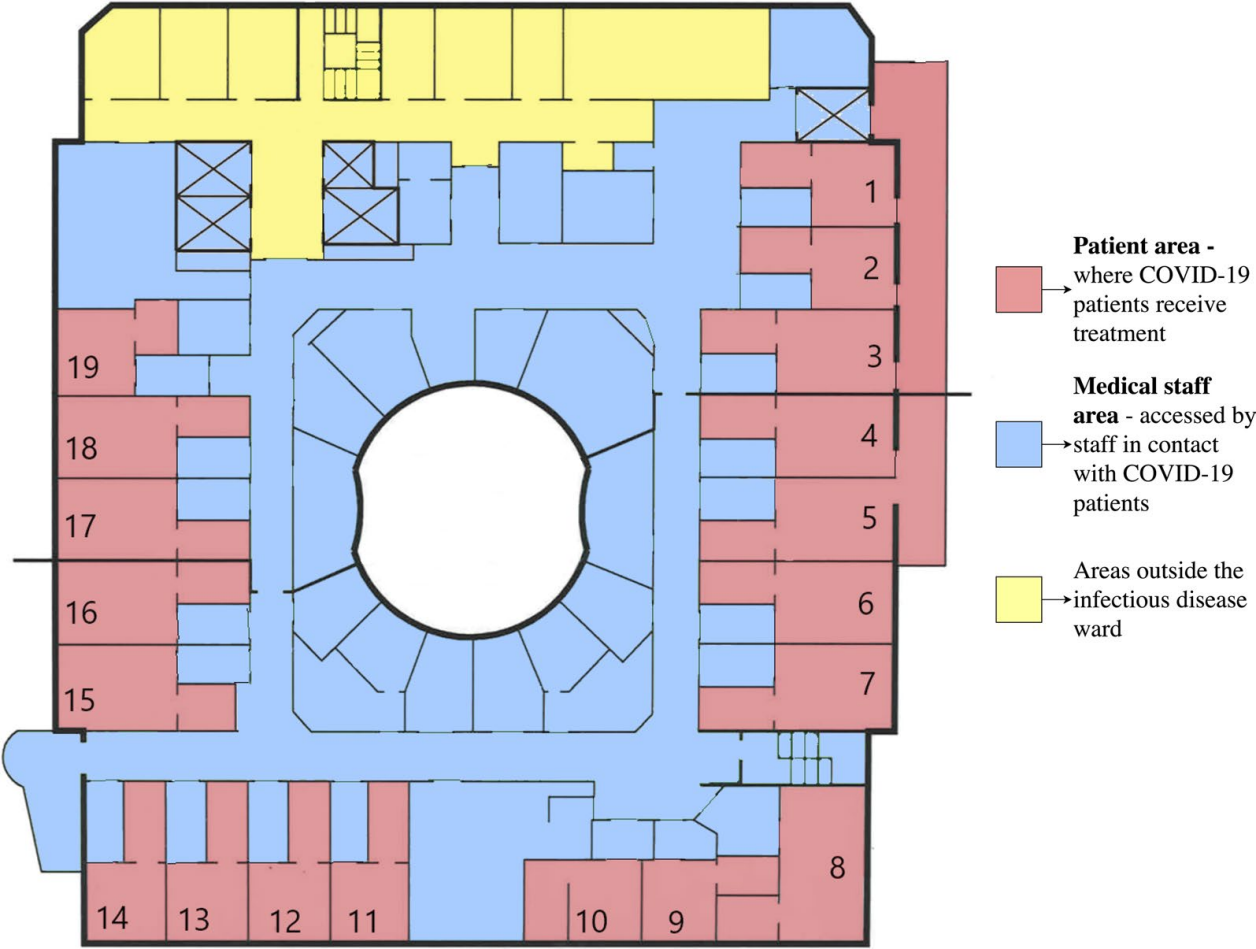
Sampling areas at the Infectious Disease ward at the Uppsala University Hospital

**Fig. 2:**
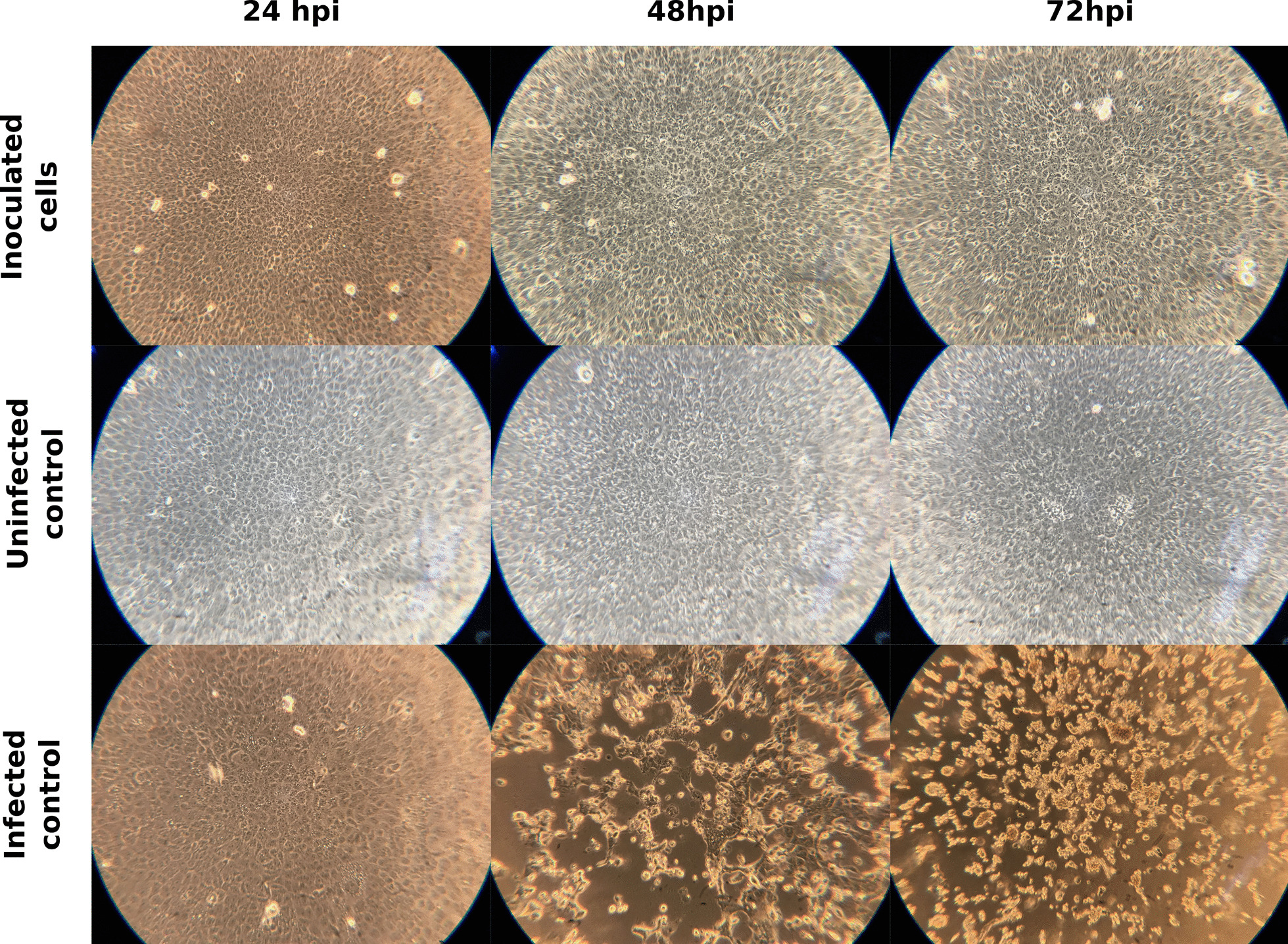
Cell CPE development in inculcated Vero E6 cells. Comparison of the CPE development between cells inoculated with the collected samples, uninfected controls and SARS-CoV-2 infected controls at 24, 48 and 72 h post infection (hpi). The shown wells are representative for all replicates, the total magnification used to observe the cells was 100x

**Fig. 3:**
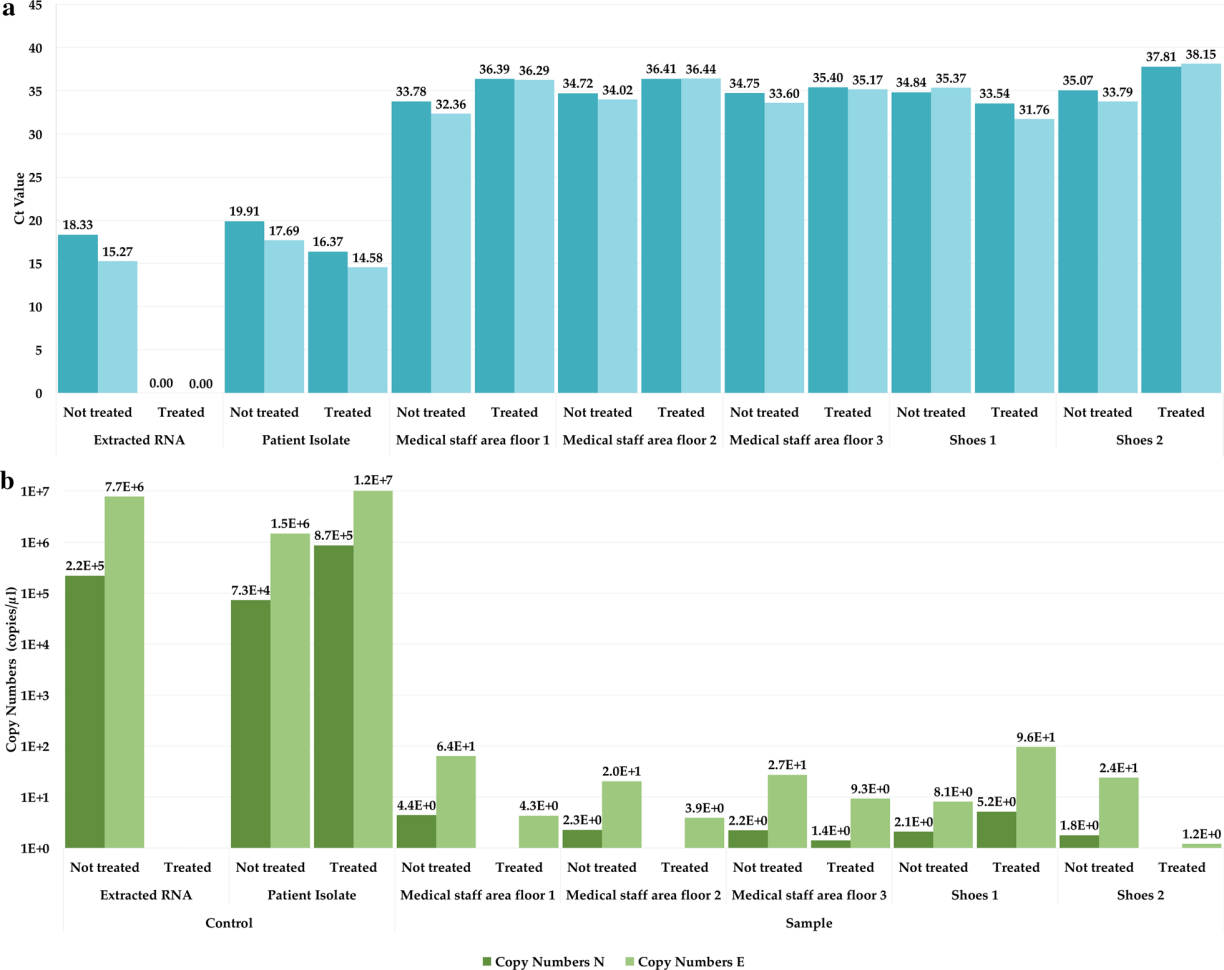
The effects of RNase treatment on the collected environmental samples in the Infectious Disease ward at the Uppsala University Hospital. **a** The difference in Ct values before and after RNase treatment as well as **b** the change in detected SARS-CoV-2 copy numbers is displayed. Individual values for N and E gene Ct values and copy numbers are given on top of the columns. Three floor swabs samples and two shoe samples were selected (based on low Ct values) for the analysis. The positive control was an infectious patient isolate of SARS-CoV-2, isolated in our laboratory, and the negative control the extracted isolate RNA. No significant change in Ct value or copy numbers was observed in the infectious virus control, corresponding to the presence of intact nucleocapsids or whole virus particles inhibiting the RNA decay. All analyzed hospital collected samples did likewise not show any significant change in Ct value or copy numbers, indicating protection of the RNA against degradation. The RNA control however was completely degraded after RNase A treatment

The sampling points were divided into five different categories: (1) patient areas where COVID-19 patients received treatment—including the patient room, patient bathroom, and ventilation openings in the patient rooms (Fig. 1); (2) medical staff areas, to which only staff in contact with COVID-19 patients had access—including anterooms, break rooms, a staff kitchen, staff computers, and the ward corridor (Fig. 1); (3) personal protective equipment—including aprons and face shields used by medical staff in patient rooms; (4) shoes—footwear used in patient rooms and medical staff areas; and (5) the ventilation system—including central ventilation ducts where air from wards, patient rooms, and medical staff areas was collected and filtered.

Three types of samples were collected: (1) swab samples for inoculation of cells and quantification of SARS-CoV-2 RNA on surfaces; (2) air samples collected with an ionization device described previously [26, 27], to assess the presence of the virus in patient room air; and (3) segments of F7 high-efficiency particulate air (HEPA) filters to assess the spread of the virus through central ventilation systems. Sampling was performed on four different occasions between early April and mid-May 2020. The relative indoor humidity ranged between 30 and 31% and the temperature between 20 and 21 °C.

### Surface swabs

Surfaces were swabbed using sterile nylon flocked swabs (Copan eSwab, Copan Italia SpA, Brescia, Italy) soaked in Virus Transport Medium (VTM) [28] containing Hank’s balanced salt solution (Gibco™, Thermo Fisher Scientific, Waltham, MA, USA) supplemented with 2% fetal bovine serum (FBS) (Gibco™, Thermo Fisher Scientific, Waltham, MA, USA), 100 µg/milliliter (µg/ml) Gentamicin (Gibco™, Thermo Fisher Scientific, Waltham, MA, USA), and 0.5 µg/ml Amphotericin B (Gibco™, Thermo Fisher Scientific, Waltham, MA, USA). The swabs were moved back and forth over an estimate area of 25 cm^2^. Surface swabs were stored in 700 µl VTM immediately after swabbing, without intermediate processing or freezing, at 4 °C for up to 24 h (h) until analysis.

### Air samples

For the collection of air sample, negative ionization was generated in an electrical field by a previously described ionization device operating at 12 V (V) [26, 27]. The generated ions collide with aerosol particles, and the subsequently negatively charged particles are collected by a collector plate with a low current of 80 microamperes (µA). The ionizer accelerates a voltage of more than 200,000 electron volts (eV). The collector plate was then repeatedly washed with 700 µl (µl) VTM and the liquids were stored at 4 °C without intermediate processing or freezing for up to 72 h until further analysis.

### Central ventilation samples

To estimate the presence of SARS-CoV-2 RNA in the central ventilation system, three 9-square-centimeter (cm^2^) sections were cut from one of six laminate F7 HEPA filters at the end of each of the three central ventilation duct systems studied and then stored in 2.5 ml VTM at 4 °C for up to 72 h without intermediate processing or freezing until analysis [17].

### RT-qPCR assay

RNA was extracted using the QIAamp viral RNA kit (Qiagen, Hilden, Germany), according to the manufacturer’s protocol and a sample volume of 280 µl. Portions of the SARS-CoV-2 envelope small membrane protein (E) and nucleocapsid (N) genes were amplified by RT-qPCR, using primers (Thermo Fisher Scientific, Waltham, MA, USA) described previously and the SuperScript™ III OneStep RT-PCR System with Platinum™ *Taq* DNA Polymerase kit (Invitrogen, Thermo Fisher Scientific, Waltham, MA, USA). Target 1 (E) [29]: forward primer 5′-ACAGGTACGTTAATAGTTAATAGCGT-3′; reverse primer 5′-TGTGTGCGTACTGCTGCAATAT-3′; and the probe 5′-FAM-ACACTAGCCATCCTTACTGCGCTTCG-TAMRA-3′. Target 2 (N) [30]: forward primer 5′-GGGGAACTTCTCCTGCTAGAAT-3′; reverse primer 5′-CAGCTTGAGAGCAAAATGTCTG-3′; and the probe 5′-FAM-TTGCTGCTGCTTGACAGATT-TAMRA-3′. The reaction mixture contained 12.5 μl of reaction buffer (a buffer containing 0.4 mM (mM) of each dNTP, 3.2 mM MgSO_4_), 0.5 μl of SuperScript™ III RT/Platinum™ Taq Mix, 0.5 μl of each primer (10 micromolar (µM) stock concentrations), 0.25 µl probe (10 µM stock concentration), 2.4 μl of 25 mM magnesium sulfate, 3.35 µL of nuclease-free water, and 5 μl of RNA template. The RT-qPCR assay was performed on a CFX96 Touch™ Real-Time PCR Detection System (Bio-Rad Laboratories, Hercules CA, USA) under the following conditions: reverse transcription at 55 °C for 30 min (min) and 95 °C for 3 min, followed by 45 cycles of denaturation at 95 °C for 15 s (s), extension at 57 °C for 30 s, and collecting the fluorescence signal at 68 °C for 30 s. A cycle threshold (Ct) value less than 40 was defined as a positive test result. Detailed RT-qPCR validation results can be found in the additional material (Additional File 1: Table S1).

All samples were run as single-runs in two different reactions for the E and N genes. The corresponding number of copies at each Ct was calculated from two standard curves prepared with synthetic DNA gene fragments (gBLOCKs; IDT®, San Jose, CA, USA) with a five-base-pair deletion in the amplified regions of the viral genome diluted in deionized, nuclease-free water to concentrations of 10^–1^ to 10^6^ copies per µl (Additional File 1: Figure S1). The five-base-pairs were deleted to be able to distinguish between viral RNA and gBLOCKs during sequencing. Triplicates of serially diluted gBLOCKs were run and the limit of detection (LOD) was determined as the lowest concentration at which all three replicates could be detected. The LODs for both genes were 10^1^ copies per µl (Additional File 1: Table S1). The relative fluorescence unit (RFU) data were obtained from the CFX Maestro™ Software ((Bio-Rad CFX Maestro for Mac 1.1 Version 4.1.2434.0214) Bio-Rad Laboratories, Hercules CA, USA).

### Virus isolation

Vero E6 cells (green monkey kidney cells (ATCC® CRL-1586™)) seeded in 6-well plates and T75 flasks in Dulbecco's Modified Eagle Medium (DMEM) (Gibco™, Thermo Fisher Scientific, Waltham, MA, USA) supplemented with 10% FBS (Gibco™, Thermo Fisher Scientific, Waltham, MA, USA) were inoculated at 90% confluency. Cells were infected with RT-qPCR positive VTM pools each containing 100 to 200 µl VTM of respective swab samples. Pooling was done according to similar collection site. Samples were stored at 4 °C for no longer than 72 h prior to infection. DMEM supplemented with 2% FBS was added, and the cell cytopathic effect (CPE) was observed daily for up to 168 h post-infection (hpi). Five hundred microliter supernatant from each pool was applied twice to uninfected cells after 168 h of respective incubation, resulting in two passages. Positive control wells were infected with SARS-CoV-2 isolated from a Swedish patient [3] at a multiplicity of infection (MOI) of 0.1. All experiments with live virus were performed inside the biosafety level three laboratory of Professor Åke Lundkvist at the Department of Medical Biochemistry and Microbiology at Uppsala University authorized by the Swedish Work Environment Authority (permit number #2016/032445).

### RNase treatment

Ten microliters of RNase A (Thermo Scientific™, Thermo Fisher Scientific, Waltham, MA, USA) were separately added to 100 µl of selected samples with low Ct values, and to the controls. SARS-CoV-2 virus isolated in our lab [3] and extracted SARS-CoV-2 RNA, were used as controls. Reactions were incubated for 45 min at room temperature and subsequently extracted and analyzed by RT-qPCR as described above.

## Results

Fifty of 200 samples (25%) collected on different surfaces in different areas of the Infectious Disease ward at the Uppsala University Hospital, Sweden, were found positive (Ct value below 40) for SARS-CoV-2 RNA by the RT-qPCR assays for both tested genes (Table 1). Another 18% of the samples were positive for one of the two tested genes. Fifty-seven percent tested negative for both genes (Ct value higher than 40 or no fluorescent signal detected).

**Table 1:** Swabs taken at different areas of the Infectious Disease ward at the Uppsala University Hospital, Sweden

Area	Negative	Positive	Positive in one gene	Total swabs
Medical staff area	68.2%	13.6%	18.2%	22
Floor	25.0%	37.5%	37.5%	8
Computer	83.3%	0.0%	16.7%	6
Door handle	100.0%	0.0%	0.0%	8
Patient area	58.8%	22.7%	18.5%	119
Bed	66.7%	33.3%	0.0%	3
Floor	43.5%	30.4%	26.1%	46
Ventilation	56.0%	24.0%	20.0%	50
Air	100.0%	0.0%	0.0%	10
Bathroom	100.0%	0.0%	0.0%	7
Door handle	100.0%	0.0%	0.0%	3
Protective gear	60.0%	40.0%	0.0%	5
Shoes	52.5%	25.0%	22.5%	40
Ventilation	35.7%	57.1%	7.1%	14
Grand total	57.0%	25.0%	18.0%	200

The number of swabs taken at different areas of the ward and the percentage of negative and positive (positive for both or one of the genes) swabs by RT-qPCRs targeting portions of the SARS-CoV-2 envelope small membrane protein and nucleocapsid genes. For areas with swabs taken in different subareas, the subcategories are given. The swabs were collected at the Infectious Disease ward at the Uppsala University Hospital, Sweden between early April and mid-May 2020

The floor, ventilation system, personal protective equipment, staff shoes, and one of the three patient beds tested positive for SARS-CoV-2 RNA. In addition, a staff computer tested positive for SARS-CoV-2 RNA in one tested gene. Swabs from door handles in patient rooms and areas for medical staff, as well as swabs from various objects in the patient bathrooms, did not show any presence of detectable SARS-CoV-2 RNA. A table with all individual Ct values can be found in the additional material (Additional File 1: Table S2).

All attempts to isolate the virus from the collected samples were unsuccessful. Combined VTM from similar sites was used to inoculate Vero E6 cells, but no cell CPE was observed (Fig. 2), nor could viral replication be detected by RT-qPCR. Likewise, two successive passages of the samples did not yield any positive results.

To determine whether positive swabs contained free SARS-CoV-2 RNA or intact virus particles, selected samples with low Ct values were treated with RNase A prior to extraction. RNase A is a nuclease, catalyzing the degradation of RNA into smaller components. The enzyme cannot surpass the viral envelope and therefore only degrades free RNA. In a comparison of Ct values before (M_N_ = 34.63, SD_N _= 0.46/M_E_ = 33.83 SD_E_ = 1.08) and after RNase digestion (M_N_ = 35.91, SD_N_ = 1.58/M_E_ = 35.56, SD_E_ = 2.38), all treated swab samples showed only a slight increase in Ct value and a corresponding slight decrease in copy number (Fig. 3), indicating the presence of intact viral nucleocapsids or whole virus particles in the samples taken from the hospital environment and not free SARS-CoV-2 RNA (t(4)_N_ = − 1.73 *P*_N_ = 0.12/ t(4)_E_ = − 1.48 *P*_*E*_ = 0.18). When RNase A was added to the extracted SARS-CoV-2 RNA from the selected hospital swabs, complete RNA digestion was determined, as no amplification was detected in the RT-qPCR assay. The RNA control was completely degraded after RNase A treatment, whereas the infectious virus control showed no significant change in Ct value (∆Ct ≈ 18%), corresponding to the presence of intact nucleocapsids or whole virus particles inhibiting the RNA decay.

## Discussion

Understanding the transmission pathways of SARS-CoV-2 is the key to limiting the spread of the virus. The community and the caregivers must be aware of how, where and when the risk of infection is greatest. Our results show that the greatest amount of SARS-CoV-2 positive swabs was observed on the floor, personal protective equipment and in the ventilation system, while no contamination was found on objects in the patient bathrooms and on door handles in various areas of the ward. The contamination of the floor and shoes could be due to respiratory droplets that fell to the floor and spread to other areas of the ward through attaching to the medical staff shoes, but droplets cannot explain the contamination of the ventilation system, indicating that the virus is transported in aerosol form. The ventilation openings are located more than two meters from the patient beds and on the ceiling. Respiratory droplets (> 5 µm) cannot drift upwards to the ventilation openings, but are affected by gravity and fall to the floor in closer proximity to the patient.

Our results demonstrate that even at moderate concentrations of viral RNA (Ct values between 30 and 38) in the collected samples no in vitro infectivity could be detected. On average, about 240 to 480 virus copies were inoculated per sample, which would be sufficient for infection according to the estimated infectious dose [31]. Since the samples were combined before inoculation, we assume that the total number of SARS-CoV-2 copy numbers in each pool was about 2,000 to 5,000.

Maintenance of viral infectiveness outside the host is crucial for possible indirect transmission via fomites. For many viral infections (i.e., caused by the severe acute respiratory syndrome coronavirus (SARS-CoV), Middle East respiratory syndrome coronavirus (MERS-CoV), influenza virus, Ebola virus and Zika virus) it is known that viral RNA can be detected in patient samples long after virus isolation is possible [32–37]. Further studies have investigated the environmental stability and infectivity of viruses from different families, such as coronaviruses (HCoV-229E, SARS-CoV, MERS-CoV and SARS-CoV-2) and bunyaviruses (Hantaan orthohantavirus, Sandfly fever Sicilian virus, Crimean-Congo hemorrhagic fever orthonairovirus and Puumala orthohantavirus) [38–44]. The majority of the investigated viruses showed the potential for stability and survival outside the host, however significant differences between various viruses were observed depending on the applied conditions and the surface material investigated. The SARS-CoV-2 virus has been shown to remain infectious for up to 28 days on stainless steel and for 74–96 h on plastic [13, 16, 18], the predominant materials investigated in this study. Another study showed that the aerosolized SARS-CoV-2 virus retained infective capability for up to 16 h at room temperature [45]. The studies discussed above showed that SARS-CoV-2 virus can survive and remain infectious on the surfaces analyzed and under the studied environmental conditions, indicating that a major part of the virus collected in our study most likely was not infectious initially.

The samples were taken in the vicinity of patients with active infections, suggesting continuous excretion of the virus in occupied rooms. It is therefore assumed that at least parts of the virions collected in the swab had been in the environment outside the patient for less than 74 h before sampling. According to the virus survival studies discussed above, the virions would likely have been able to retain their infectivity if they had been infectious when they left the patient. The lack of infectivity of the detected virions is therefore assumed to be a consequence of the fact that a major part of the virions was likely not infectious at the time of surface contamination.

We were able to establish that the detected contamination was presumably due to intact nucleocapsids or whole virions and not due to free viral RNA. Comparing results from the RT-qPCR pre (M_N_ = 34.63, SD_N _= 0.46/M_E_ = 33.83 SD_E_ = 1.08) and post (M_N_ = 35.91, SD_N_ = 1.58/M_E_ = 35.56, SD_E_ = 2.38) RNase treatment of the samples indicated that that the viral RNA was protected against enzyme degradation, t(4)_N_ = − 1.73 *P*_N_ = 0.12/ t(4)_E_ = − 1.48 *P*_E_ = 0.18. After RNase A digestion and subsequent RNA extraction, the analyzed samples showed no significant change in the Ct value as compared to the pre-treatment extraction (*P* > 0.05) and the corresponding copy numbers. The only small variation in the Ct value and corresponding copy numbers of the SARS-CoV-2 patient isolate as well as the complete eradication of free SARS-CoV-2 RNA after digestion show the suitability of the chosen RNase A digestion method for the intact nucleocapsid or virions detection. The RNase cannot surpass the viral envelope nor the nucleocapsid and therefore only degrades free RNA. Therefore, the extracted RNA control is expected to be fully degraded whereas the intact virions/nucleocapsids in the patient samples will inhibit the RNase from reaching the viral RNA.

## Conclusion

Our findings indicate that the SARS-CoV-2 virus exists in two forms outside the host; as infectious and as non-infectious. As supported by previous studies it appears that symptomatic patients in the hospital stop shedding infectious virus particles after a few days but continue shedding non-infectious virus particles [20–23]. The potential reasons for the observed absence of infective capability remain to be investigated. Possible explanations include an intact viral envelope but damaged surface proteins and/or the virions being covered by neutralizing antibodies (likely IgA) and/or damaged envelopes but protective nucleocapsids, all leading to the inhibition of viral cell entry. The prolonged duration of successful virus isolation described in immunocompromised patients condition (24) supports the assumption that the patient's immune system could be involved in causing the virus to lose its ability to infect. We believe that the results of our study will have an impact on the determination of the risk of healthcare workers, especially for those working with patients in earlier stages of the infection with a higher likelihood of the patients being shedders of infectious virus particles.

Data on the duration of virus shedding in correlation to infectivity of hospital admitted patients and outpatients, are urgently needed to further enhance infection prevention.

## Supplementary Information

**Additional File 1.** RT-qPCR validation results and individual Ct values.

## Data Availability

The dataset supporting the conclusions of this article is included within the article (and its additional file). The additional file contains: Additional File 1: Figure S1: Standard curves generated with synthetic DNA gene fragments; Additional File 1: Table S1: RT-qPCR validation results; Additional File 1: Table S2: Ct values for all collected samples at the Uppsala University infectious disease ward.

## Abbreviations

COVID-19: Coronavirus disease
CPE: Cell cytopathic effect
Ct: Cycle threshold
DMEM: Dulbecco's modified eagle medium
DNA: Desoxyribonucleic acid
FBS: Fetal bovine serum
HEPA: High-efficiency particulate air
Hpi: Hours post infection
LOD: Limit of detection
MERS-CoV: Middle East respiratory syndrome coronavirus
MOI: Multiplicity of infection
MgSO_4_: Magnesium sulfate
NTPs: Nucleoside triphosphates
RFU: Relative fluorescence units
RNA: Ribonucleic acid
RT-qPCR: Reverse transcription quantitative polymerase chain reaction
SARS-CoV: Severe acute respiratory syndrome coronavirus
SARS-CoV-2: Severe acute respiratory syndrome coronavirus 2
VTM: Virus transport medium
