# Supplementary material for: SARS-CoV-2 in hospital indoor environments is predominantly non-infectious
Source: Virol J. 2021 Jun 2;18:109. doi: 10.1186/s12985-021-01556-6 (PMC8170062; doi:10.1186/s12985-021-01556-6)
Supplement: Supplementary file 1 — Additional File 1. RT-qPCR validation results and individual Ct values. [file 12985_2021_1556_MOESM1_ESM.pdf]

Supplementary Materials

SARS-CoV-2 in hospital indoor environments is predominantly non-infectious

Janina Krambrich, Dario Akaberi, Jiaxin Ling, Tove Hoffman, Lennart Svensson, Marie Hagbom, Åke Lundkvist

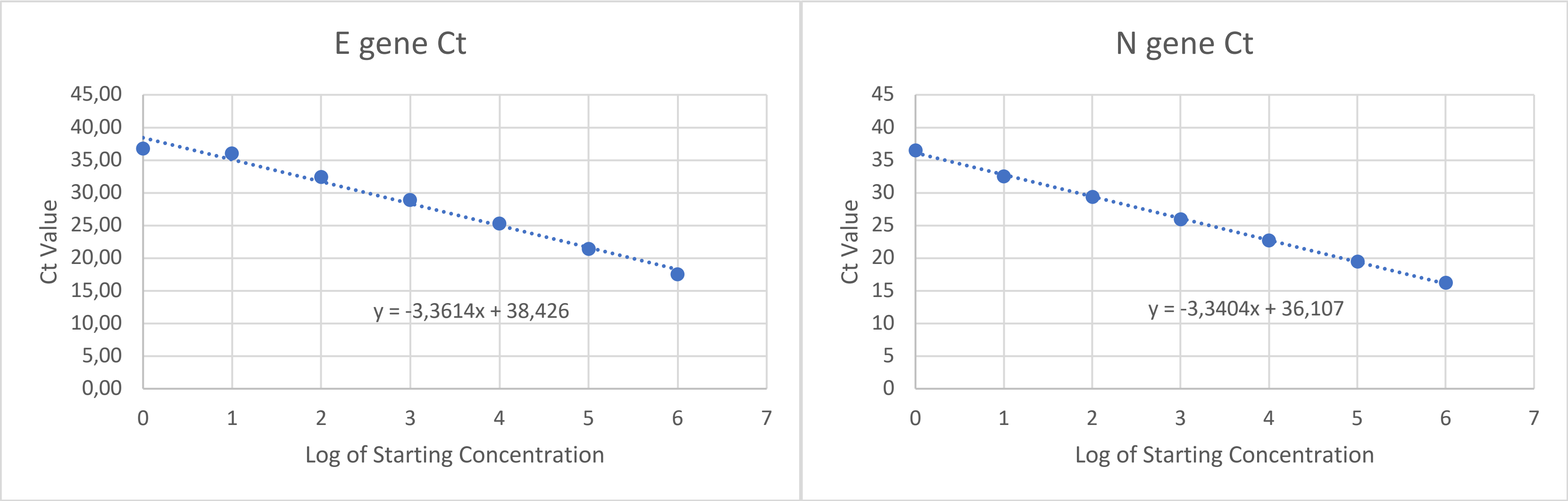

Figure S1: Standard curves generated with synthetic DNA gene fragments for correlating of copy numbers and Ct values
Standard curves generated with synthetic DNA gene fragments (gBLOCKs; IDT®, San Jose, CA, USA) with a five base-pair deletion in the amplified regions of the viral genome diluted in deionized, nuclease-free water to concentrations of 10^{-1} to 10^{6} copies per µl. The linear equation can be used to calculate the corresponding copy numbers for Ct values.

Table S1: RT-qPCR validation results.

	**E gene**	**N gene**
**Limit of detection (LOD)**	10^{1} copies/µl	10^{1} copies/µl
**Efficiency (E)**	101.5%	103.1%
**Coefficient of determination (R2)**	0.987	0.998
**Linear dynamic range (LDR)**	10^{6} to 10^{-1} copies/µl	10^{6} to 10^{-1} copies/µl
**Specificity**	Determined by amplicon size visualized through gel electrophoresis and by sequencing	

Validation result for the used SARS-CoV-2 RT-qPCR assay.

Table S2: Ct values for all collected samples at the Uppsala University infectious disease ward

RT-qPCR results for all swabs taken at the Akademiska Hospital Infectious Disease ward in Uppsala, Sweden

Sample group	Sub group	RT-qPCR results		Sample group	Sub group	RT-qPCR results		Sample group	Sub group	RT-qPCR results		Sample group	Sub group	RT-qPCR results	
		N gene	E gene			N gene	E gene			N gene	E gene			N gene	E gene
Medical staff area	Computer	-	37.78	Patient area	Floor	33.78	32.36	Patient area	Ventilation	37.26	36.14	Shoes	Shoes	38.18	-
Medical staff area	Computer	-	-	Patient area	Floor	34.72	34.02	Patient area	Ventilation	37.56	35.28	Shoes	Shoes	38.70	-
Medical staff area	Computer	-	-	Patient area	Floor	34.75	33.60	Patient area	Ventilation	37.94	37.90	Shoes	Shoes	38.71	-
Medical staff area	Computer	-	-	Patient area	Floor	36.37	35.12	Patient area	Ventilation	38.61	37.55	Shoes	Shoes	39.76	-
Medical staff area	Computer	-	-	Patient area	Floor	37.09	36.90	Patient area	Ventilation	38.75	38.45	Shoes	Shoes	34.84	35.37
Medical staff area	Computer	-	-	Patient area	Floor	37.24	34.14	Patient area	Ventilation	38.82	37.26	Shoes	Shoes	35.07	33.79
Medical staff area	Floor	36.00	35.46	Patient area	Floor	37.28	36.67	Patient area	Ventilation	39.55	38.71	Shoes	Shoes	35.27	35.09
Medical staff area	Floor	36.60	37.81	Patient area	Floor	37.62	36.29	Patient area	Ventilation	39.77	38.95	Shoes	Shoes	36.16	34.87
Medical staff area	Floor	38.60	36.26	Patient area	Floor	38.31	35.85	Patient area	Ventilation	-	39.83	Shoes	Shoes	36.76	36.75
Medical staff area	Floor	37.64	-	Patient area	Floor	38.61	35.64	Patient area	Ventilation	-	38.74	Shoes	Shoes	37.73	36.52
Medical staff area	Floor	38.02	-	Patient area	Floor	38.86	36.64	Patient area	Ventilation	-	38.63	Shoes	Shoes	37.74	36.52
Medical staff area	Floor	38.06	-	Patient area	Floor	38.97	37.82	Patient area	Ventilation	-	37.76	Shoes	Shoes	38.00	36.68
Medical staff area	Floor	-	-	Patient area	Floor	39.22	34.39	Patient area	Ventilation	-	36.78	Shoes	Shoes	38.76	36.73
Medical staff area	Floor	-	-	Patient area	Floor	-	37.86	Patient area	Ventilation	-	-	Shoes	Shoes	38.77	36.23
Medical staff area	Door handle	-	-	Patient area	Floor	-	37.77	Patient area	Ventilation	-	-	Shoes	Shoes	-	36.55
Medical staff area	Door handle	-	-	Patient area	Floor	-	37.21	Patient area	Ventilation	-	-	Shoes	Shoes	-	35.37
Medical staff area	Door handle	-	-	Patient area	Floor	-	36.70	Patient area	Ventilation	-	-	Shoes	Shoes	-	-
Medical staff area	Door handle	-	-	Patient area	Floor	-	36.54	Patient area	Ventilation	-	-	Shoes	Shoes	-	-
Medical staff area	Door handle	-	-	Patient area	Floor	-	-	Patient area	Ventilation	-	-	Shoes	Shoes	-	-
Medical staff area	Door handle	-	-	Patient area	Floor	-	-	Patient area	Ventilation	-	-	Shoes	Shoes	-	-
Medical staff area	Door handle	-	-	Patient area	Floor	-	-	Patient area	Ventilation	-	-	Shoes	Shoes	-	-
Medical staff area	Door handle	-	-	Patient area	Floor	-	-	Patient area	Ventilation	-	-	Shoes	Shoes	-	-
Patient area	Air	-	-	Patient area	Floor	-	-	Patient area	Ventilation	-	-	Shoes	Shoes	-	-
Patient area	Air	-	-	Patient area	Floor	-	-	Patient area	Ventilation	-	-	Shoes	Shoes	-	-
Patient area	Air	-	-	Patient area	Floor	-	-	Patient area	Ventilation	-	-	Shoes	Shoes	-	-
Patient area	Air	-	-	Patient area	Floor	-	-	Patient area	Ventilation	-	-	Shoes	Shoes	-	-
Patient area	Air	-	-	Patient area	Floor	-	-	Patient area	Ventilation	-	-	Shoes	Shoes	-	-
Patient area	Air	-	-	Patient area	Floor	-	-	Patient area	Ventilation	-	-	Shoes	Shoes	-	-
Patient area	Air	-	-	Patient area	Floor	-	-	Patient area	Ventilation	-	-	Shoes	Shoes	-	-
Patient area	Air	-	-	Patient area	Floor	-	-	Patient area	Ventilation	-	-	Shoes	Shoes	-	-
Patient area	Air	-	-	Patient area	Floor	-	-	Patient area	Ventilation	-	-	Shoes	Shoes	-	-
Patient area	Air	-	-	Patient area	Floor	-	-	Patient area	Ventilation	-	-	Shoes	Shoes	-	-
Patient area	Bathroom	-	-	Patient area	Floor	-	-	Patient area	Ventilation	-	-	Shoes	Shoes	-	-
Patient area	Bathroom	-	-	Patient area	Floor	-	-	Patient area	Ventilation	-	-	Shoes	Shoes	-	-
Patient area	Bathroom	-	-	Patient area	Floor	-	-	Patient area	Ventilation	-	-	Shoes	Shoes	-	-

Patient area	Bathroom	-	-	Patient area	Floor	-	-	Patient area	Ventilation	-	-	Shoes	Shoes	-	-
Patient area	Bathroom	-	-	Patient area	Floor	-	-	Patient area	Ventilation	-	-	Ventilation	Ventilation	35.32	35.41
Patient area	Bathroom	-	-	Patient area	Floor	-	-	Patient area	Ventilation	-	-	Ventilation	Ventilation	35.72	33.85
Patient area	Bathroom	-	-	Patient area	Door handle	-	-	Patient area	Ventilation	-	-	Ventilation	Ventilation	36.31	34.87
Patient area	Bed	35.50	35.10	Patient area	Door handle	-	-	Patient area	Ventilation	-	-	Ventilation	Ventilation	36.72	36.08
Patient area	Bed	-	-	Patient area	Door handle	-	-	Patient area	Ventilation	-	-	Ventilation	Ventilation	36.86	34.91
Patient area	Bed	-	-	Patient area	Ventilation	39.04	-	Protective gear	Personal protective gear	37.74	35.91	Ventilation	Ventilation	37.13	37.30
Patient area	Floor	37.29	-	Patient area	Ventilation	39.28	-	Protective gear	Personal protective gear	37.98	36.73	Ventilation	Ventilation	37.42	38.70
Patient area	Floor	37.34	-	Patient area	Ventilation	39.53	-	Protective gear	Personal protective gear	-	-	Ventilation	Ventilation	38.79	36.96
Patient area	Floor	37.58	-	Patient area	Ventilation	39.78	-	Protective gear	Personal protective gear	-	-	Ventilation	Ventilation	-	38.40
Patient area	Floor	38.21	-	Patient area	Ventilation	39.95	-	Protective gear	Personal protective gear	-	-	Ventilation	Ventilation	-	-
Patient area	Floor	38.79	-	Patient area	Ventilation	35.33	33.77	Shoes	Shoes	-	-	Ventilation	Ventilation	-	-
Patient area	Floor	38.90	-	Patient area	Ventilation	36.09	33.93	Shoes	Shoes	37.31	-	Ventilation	Ventilation	-	-
Patient area	Floor	38.93	-	Patient area	Ventilation	36.24	36.70	Shoes	Shoes	37.48	-	Ventilation	Ventilation	-	-
Patient area	Floor	32.81	30.57	Patient area	Ventilation	36.78	35.31	Shoes	Shoes	37.97	-	Ventilation	Ventilation	-	-

Medical staff areas, to which only staff in contact with COVID-19 patients had access – including anterooms, break rooms, a staff kitchen, staff computers, and the ward corridor; Patient areas where COVID-19 patients received treatment - including the patient room, patient bathroom, and ventilation openings in the patient rooms; Personal protective equipment – including aprons and face shields used by medical staff in patient rooms; Shoes – footwear used in patient rooms and in medical staff areas; and the Ventilation system – including central ventilation ducts where air from wards, patient rooms, and medical staff areas was collected and filtered
